# Unveiling global research trends and hotspots on mitochondria in NAFLD from 2000 to 2023: A bibliometric analysis

**DOI:** 10.1002/iid3.1226

**Published:** 2024-03-27

**Authors:** Jingqin Hu, Ze Chen, Yibing Zhou, Yinglun Li, Jing Liu, Yuqiang Mi, Li Wang, Feng Jiang, Ping Li

**Affiliations:** ^1^ Clinical School of the Second People's Hospital Tianjin Medical University Tianjin China; ^2^ Department of Hepatology Tianjin Second People's Hospital Tianjin China; ^3^ Department of Pharmacy Tianjin Second People's Hospital Tianjin China; ^4^ Department of Neonatology Obstetrics and Gynecology Hospital of Fudan University Shanghai China

**Keywords:** bibliometrics, CiteSpace, mitochondria, NAFLD, VOSviewers

## Abstract

**Background:**

Nonalcoholic fatty liver disease (NAFLD) has garnered significant attention in the past decade as a prevalent chronic liver condition. Despite a growing body of evidence implicating mitochondria in NAFLD development, comprehensive bibliometric analyses within this research domain are scarce. This study aims to provide a thorough overview of the knowledge framework and key research areas related to mitochondria in the context of NAFLD, utilizing bibliometric techniques.

**Methods:**

A comprehensive search of publications on mitochondria in NAFLD from 2000 to 2023 was conducted using the Web of Science Core Collection database. VOSviewers, CiteSpace, and the R package “bibliometrix” were employed for a precise assessment of the literature.

**Results:**

Examining 2530 articles from 77 countries, primarily led by the United States and China, revealed a consistent increase in publications on mitochondria's role in NAFLD. Leading research institutions include the University of Coimbra, the University of Missouri, the Chinese Academy of Sciences, Fudan University, and Shanghai Jiao Tong University. Notably, the *International Journal of Molecular Sciences* emerged as the most popular journal, and *Hepatology* was the most frequently cited. With contributions from 14,543 authors, Michael Roden published the highest number of papers, and A. J. Samyal was the most frequently cocited author. Key focus areas include investigating mitochondrial mechanisms impacting NAFLD and developing therapeutic strategies targeting mitochondria. Emerging research hotspots are associated with keywords such as “inflammation,” “mitochondrial dysfunction,” “autophagy,” “obesity,” and “insulin resistance.”

**Conclusion:**

This study, the first comprehensive bibliometric analysis, synthesizes research trends and advancements in the role of mitochondria in NAFLD. Insights derived from this analysis illuminate current frontiers and emerging areas of interest, providing a valuable reference for scholars dedicated to mitochondrial studies.

## 1 INTRODUCTION

Nonalcoholic fatty liver disease (NAFLD) is a prevalent condition that affects the liver worldwide. It is a chronic liver disease characterized by the accumulation of lipids, inflammation, and insulin resistance (IR).^1^ NAFLD is closely linked to obesity, cardiovascular disease, and metabolic syndrome. If left untreated, it can progress to more severe conditions such as nonalcoholic steatohepatitis (NASH), liver fibrosis, cirrhosis, and liver malignancies.^2^, ^3^ However, despite its significant impact, our understanding of the underlying causes and progression of NAFLD remains incomplete.^4^ Therefore, it is of utmost importance to conduct a comprehensive examination of the historical evolution and current state of NAFLD research.

Mitochondria play a vital role in cellular biosynthesis, bioenergetics, and intracellular signaling, maintaining normal physiological functions.^5^ Various internal and external stressors can disrupt mitochondrial homeostasis, affecting essential processes such as the redox system, oxidative phosphorylation, mitochondrial biogenesis, and mitophagy.^6^ Recent studies have revealed that mitochondria have a significant impact on the emergence and progression of NAFLD, including mitochondrial dynamics and mitophagy that eliminate excessive reactive oxygen species (ROS), mitochondrial DNA (mtDNA), and other factors.^7^, ^8^, ^9^

Bibliometrics is a method of literature analysis that quantitatively and qualitatively assesses publications in a specific research domain.^10^ This approach provides detailed information on authors, keywords, journals, countries, institutions, and references within the relevant research area.^11^ Various bibliometric tools have gained popularity in the medical field, particularly in disciplines like oncology, respiratory and infection.^12^, ^13^, ^14^ These tools, including CiteSpace, VoSviewer, and the R package “bibliometrix,” offer valuable visualization capabilities to aid in literature analyses.^14^, ^15^, ^16^ Researchers have extensively utilized these resources to effectively present and interpret their findings. However, there is a lack of bibliometric investigations focusing on mitochondria in the context of NAFLD.

Understanding the current state, key areas of research, and prospects of mitochondria in NAFLD can reveal the relationship between knowledge structure in this field and disease progression mechanisms. To gain a better understanding of the roles of mitochondria in NAFLD and develop novel approaches for prevention and treatment, it is crucial to delve deeper into this area. This study endeavors to comprehensively examine scientific discoveries about the involvement of mitochondria in NAFLD, with the ultimate objective of offering fresh perspectives to tackle the clinical obstacles associated with prevention and treatment.

## 2 MATERIALS AND METHODS

### 2.1 Search strategy

**Figure 1:**
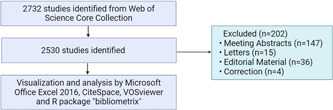
Publications screening flowchart.

A literature search was conducted on the Web of Science Core Collection (WoSCC) database (https://www.webofscience.com/wos/woscc/basic-search) on September 20, 2023, with topical retrieval terms ranging from January 1, 2000 to September 20, 2023. The search formula used was ((TS = (mitochondria OR mitochondrion OR mitochondrial))) AND ALL = (Nonalcoholic Fatty Liver Disease OR Nonalcoholic Fatty Liver Disease OR NAFLD OR Nonalcoholic Fatty Liver OR Nonalcoholic Steatohepatitis) AND LA = (English). The document type was restricted to “articles” and “reviews” (Figure 1).

### 2.2 Data analysis

VOSviewer (version 1.6.19) is a well‐known bibliometric analysis software that is highly regarded for its ability to extract valuable insights from a large number of publications.^17^ It is commonly used for constructing collaboration networks, cocitation networks, and cooccurrence networks. In our study, this software was employed to perform essential analyses, including country and institution analysis, journal and cocited journal analysis, author and cocited author analysis, and keyword cooccurrence analysis. When VOSviewer generates a visualization map, each node in the map represents a distinct entity, such as countries, institutions, journals, and authors. The size of a node corresponds to the number of items it represents, and the node's color categorizes these items into different categories. The thickness of the lines connecting nodes indicates the degree of collaboration or cocitation between these entities. This comprehensive analytical tool is a valuable resource for identifying meaningful patterns and connections within the scholarly landscape, assisting researchers in gaining deeper insights into their research fields.

CiteSpace, particularly version 6.2.R4, is a highly useful software tool developed by Professor Chen C. for conducting bibliometric analysis and visualization.^18^ In our research, we made use of CiteSpace to create dual‐map overlays of journals, as well as to carry out reference analysis with a specific emphasis on Citation Bursts. This software allows researchers to visualize the complex interaction of scientific publications and identify emerging trends and influential references.

The R package “bibliometrix” (version 4.3.1), available at https://www.bibliometrix.org, facilitated a comprehensive examination of the thematic evolution within the field and enabled the construction of a global distribution network of publications related to mitochondria in NAFLD.^19^

To complement our analysis, quartile and impact factor data for journals from the Journal Citation Reports for the year 2022 were accessed. Additionally, Microsoft Office Excel 2016 was utilized to conduct quantitative analyses of the publications, enabling the extraction of meaningful insights and the drawing of informed conclusions from the data.

## 3 RESULTS

### 3.1 Quantitative analysis of publication

**Figure 2:**
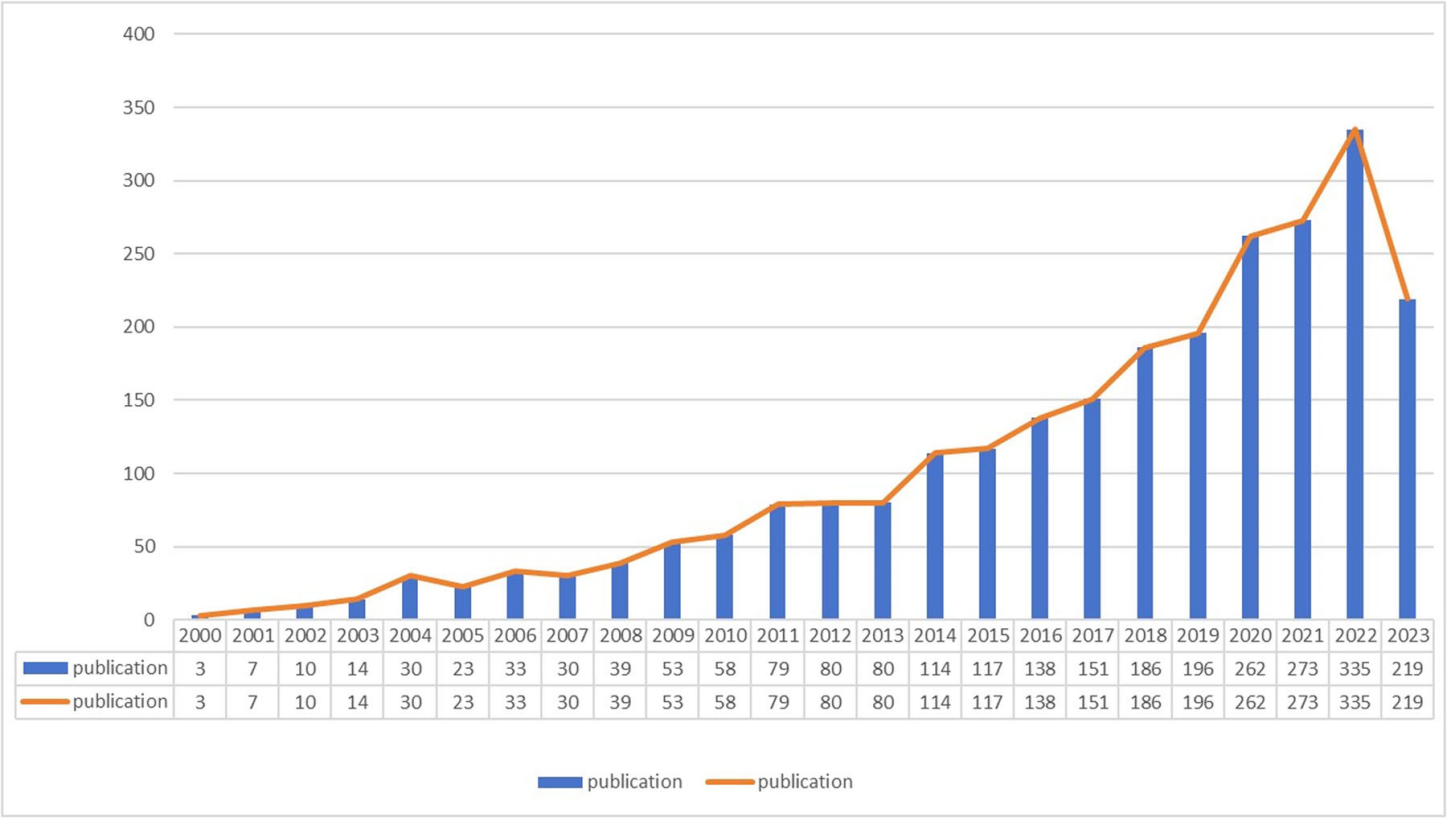
Annual output of research of mitochondria in NAFLD. NAFLD, nonalcoholic fatty liver disease.

Through our comprehensive search strategy, we have identified a substantial number of studies, totaling 2530, that investigate the crucial role of mitochondria in NAFLD. These studies encompass a range of publications, including 1884 research articles and 646 review articles. As shown in Figure 2, the frequency of these publications has exhibited a persistent upward trajectory over the past 22 years. This consistent increase in research interest and output demonstrates the enduring and growing fascination with the involvement of mitochondria in NAFLD.

During the initial phase from 2000 to 2008, the volume of publications within this field remained relatively modest. The annual publication count did not surpass 40, suggesting that this period marked the early stages of mitochondrial research concerning NAFLD. The limited number of publications during this time indicates that the relevant theories in this field had not yet received comprehensive validation. However, as time progressed, there has been a noticeable shift in focus towards mitochondria in the context of NAFLD research. This evolution signifies an increasing recognition of the central role played by mitochondria in this field and a growing interest in unraveling their intricate involvement.

From 2009 until the present, there has been a significant surge in the number of publications within this field. This trend highlights the growing scholarly interest and research activity in the study of mitochondria in NAFLD. Alongside this increase in publications, the body of research outcomes has expanded substantially. Collectively, these observations indicate that the investigation of mitochondria in NAFLD has emerged as a prominent and rapidly advancing area of study.

### 3.2 Country and institutional analysis

**Figure 3:**
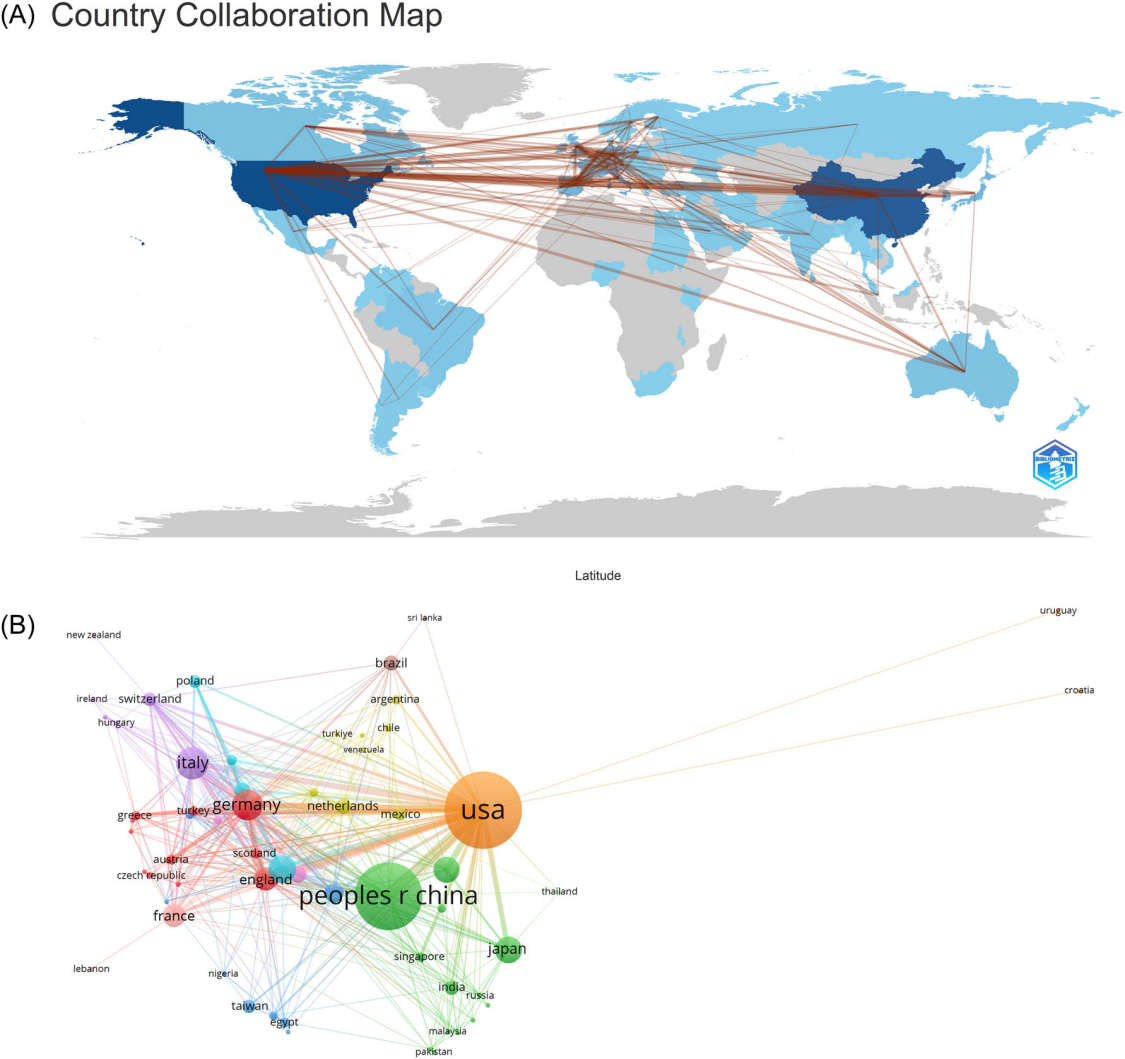
The geographical distribution (A) and visualization of countries (B) on the research of mitochondria in NAFLD. NAFLD, nonalcoholic fatty liver disease.

To investigate the global contributions to this research field, a closer look at the top 10 countries involved reveals that they are spread across Asia, North America, and Europe, with a predominant presence in Asia (*n* = 3) and Europe (*n* = 5), as shown in Table 1. Notably, the United States leads with the highest number of publications (*n* = 796, 31.5%), closely followed by China (*n* = 642, 25.4%), Italy (*n* = 192, 7.6%), and Germany (*n* = 176, 7.0%). Remarkably, the combined publications from China and the United States make up nearly half of the total output (44.7%). To comprehensively assess international collaboration within this field, we filtered and visually represented data from 63 countries that had published two or more articles. The resulting collaborative network, as depicted in Figure 3, illustrates a dynamic landscape of cooperation among these nations. For instance, China actively collaborates with the United States, Japan, and South Korea, while the United States maintains robust partnerships with Italy, Spain, and Germany.

**Table 1:** Top 10 countries and institutions on the research of mitochondria in NAFLD.

Rank	Country	Counts	Institution	Counts
1	USA	796	University of Coimbra	38
2	China	642	University of Missouri	38
3	Italy	192	Chinese Acad Sciand	33
4	Germany	176	Fudan University	33
5	Spain	143	Shanghai Jiao Tong University	33
6	Japan	141	Yale University	31
7	South Korea	135	Capital Medical University	31
8	UK	109	Huazhong University of Science and Technology	31
9	France	105	Zhejiang University	30
10	Canada	79	University of Pittsburgh	28

Abbreviation: NAFLD, nonalcoholic fatty liver disease.

Upon examination, it is evident that the top 10 institutions contributing to the field are spread across three countries. However, it is worth noting that a majority of these institutions are located in China. Out of these top 10 institutions, four of them have emerged as the most active publishers of relevant research papers. These institutions are the University of Coimbra, the University of Missouri, the Chinese Academy of Sciences, Fudan University, and Shanghai Jiao Tong University. Impressively, six out of the top 10 institutions are based in China, which highlights the significant influence that China holds in this particular domain.

**Figure 4:**
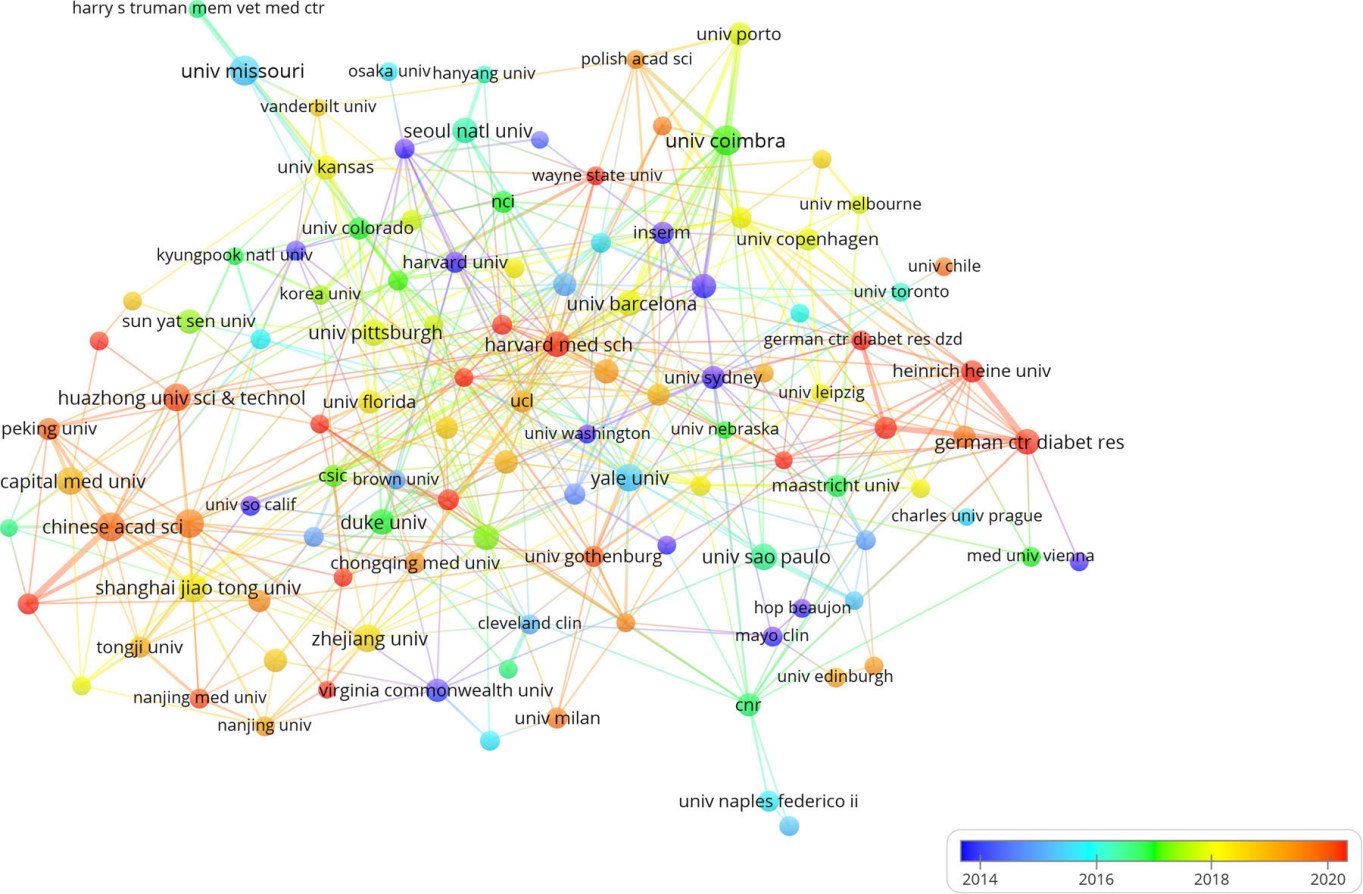
The visualization of institutions on the research of mitochondria in NAFLD. NAFLD, nonalcoholic fatty liver disease.

When exploring this landscape further, it becomes evident that the University of Coimbra, the University of Missouri, and the Chinese Academy of Sciences are the top three institutions with the highest number of publications (Np). To delve deeper into this subject, we selected 115 institutions for visualization purposes. To be included in this network, each institution had to meet a minimum publication threshold of 10. Figure 4 effectively depicts this network, revealing interesting patterns of collaboration.

Notably, a close‐knit collaboration exists among the Chinese Academy of Sciences, Peking University, and Harvard Medical School. Further active cooperation is evident between Yale University, the University of Milan, and the German Center for Diabetes Research. Additionally, it is worth mentioning that Chinese institutions primarily engage in collaborations within their own country, with a comparably lower level of international exchanges.

### 3.3 Journal and cocited journals

The field of mitochondrial studies in NAFLD has a broad research landscape, with contributions from a total of 616 journals. These journals play a vital role in disseminating knowledge within this field. One notable frontrunner in this domain is the “*International Journal of Molecular Sciences*,” which has published the highest number of papers (108) related to this topic. Following closely behind are “*Hepatology*” with 66 papers, “*Journal of Hepatology*” with 55 papers, and “*Scientific Reports*” with 46 papers. In terms of impact factor, “*Journal of Hepatology*” holds the highest position among the top 15 journals with an impressive IF of 25.70 (Q1), closely followed by “*Hepatology*” with an IF of 17.29 (Q1) (Table 2).

**Table 2:** Top 15 journals and cocited journals for research of mitochondria in NAFLD.

Rank	Journal	Count	IF	Q	Cocited journal	Cocitation	IF	Q
1	*International Journal of Molecular Sciences*	108	6.208	Q1	*Hepatology*	9270	17.298	Q1
2	*Hepatology*	66	17.298	Q1	*Cell Metabolism*	4362	28.999	Q1
3	*Journal of Hepatology*	55	25.701	Q1	*Journal of Hepatology*	4335	25.701	Q1
4	*Scientific Reports*	46	4.600	Q2	*Gastroenterology*	3881	29.400	Q1
5	*Nutrients*	45	6.706	Q1	*Metabolism‐Clinical and Experimental*	2767	9.800	Q1
6	*Antioxidants*	35	6.999	Q1	*World Journal of Gastroenterology*	2639	4.299	Q2
7	*Free Radical Biology and Medicine*	33	7.400	Q1	*Free Radical Biology and Medicine*	2290	7.400	Q1
8	*World Journal of Gastroenterology*	33	4.299	Q2	*Seminars in Liver Disease*	2283	4.199	Q2
9	*PLoS One*	33	3.700	Q2	*Nature Reviews Gastroenterology*	2106	65.100	Q1
10	*The Journal of Nutritional Biochemistry*	31	5.599	Q1	*Journal of Clinical Investigation*	2028	15.900	Q1
11	*Frontiers in Pharmacology*	31	5.599	Q1	*Journal of Biological Chemistry*	1988	4.800	Q2
12	*American Journal of Physiology‐Endocrinology and Metabolism*	30	5.100	Q1	*American Journal of Physiology‐Gastrointestinal and Liver Physiology*	1955	4.500	Q2
13	*Oxidative Medicine and Cellular Longevity*	28	7.310	Q2	*Nutrients*	1563	6.706	Q1
14	*Liver International*	27	6.700	Q1	*Journal of Gastroenterology and Hepatology*	1509	4.099	Q2
15	*Biochemical and Biophysical Research Communications*	27	3.100	Q2	*American Journal of Physiology‐Endocrinology and Metabolism*	1464	5.100	Q1

Abbreviation: NAFLD, nonalcoholic fatty liver disease.

**Figure 5:**
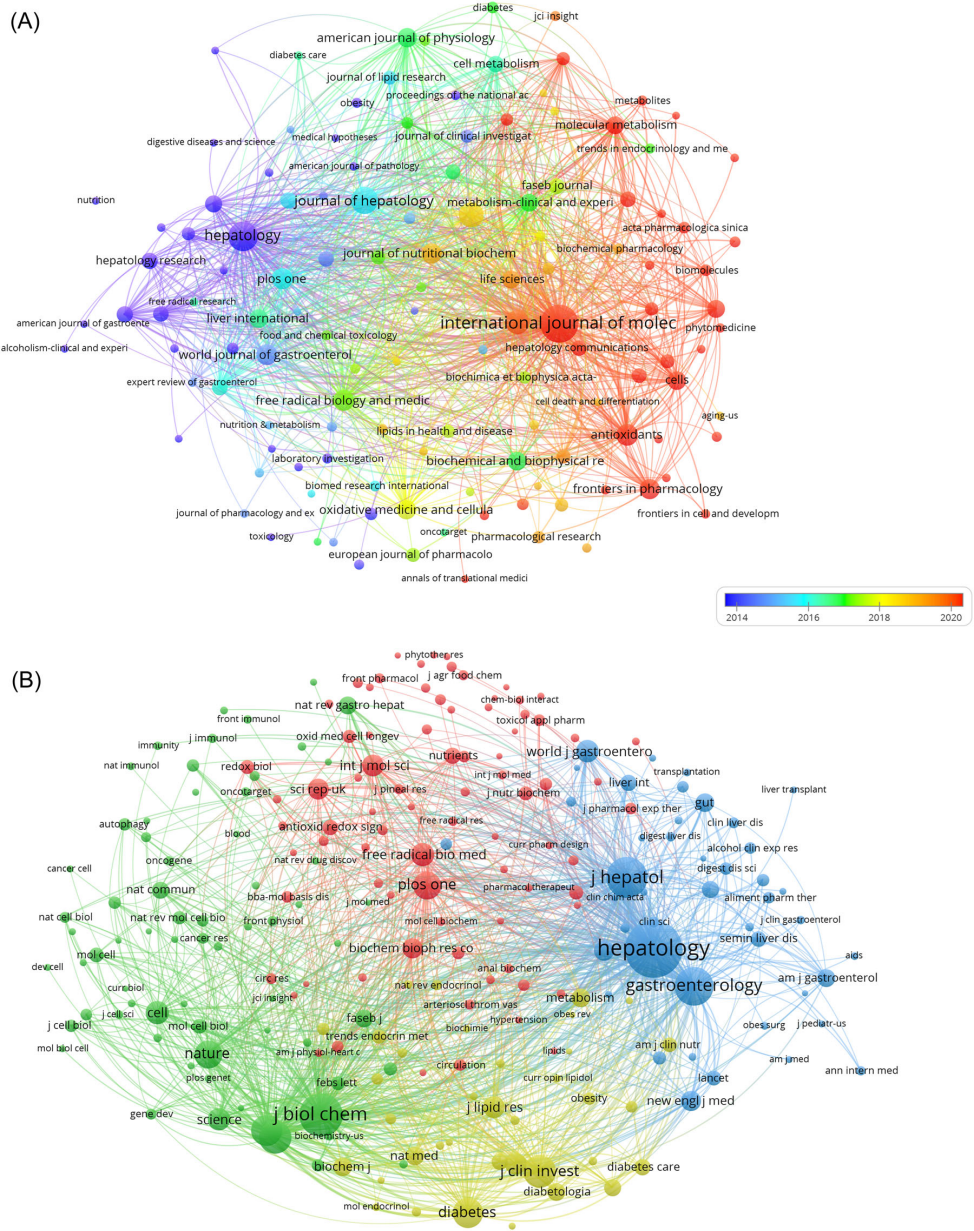
The visualization of journals (A) and cocited journals (B) on the research of mitochondria in NAFLD. NAFLD, nonalcoholic fatty liver disease.

To obtain a comprehensive overview of the relationships and citations among these journals, we conducted a systematic screening of 126 journals, applying a minimum publication threshold of five articles. The resulting journal network, depicted in Figure 5A, provides valuable insights into the interplay among these publications. Notably, the “*International Journal of Molecular Sciences*” demonstrates active citation relationships with journals such as “Hepatology,” “Biomolecules,” and “Scientific Reports,” among others.

Cocitation between two articles means that both articles appear in the reference list of a third article. Table 2 showcases the cocitation patterns of the top 15 journals within this research field. Remarkably, three journals have accumulated over 4000 cocitations, with “*Hepatology*” leading the way (cocitation = 9270), closely followed by “*Cell Metabolism*” (cocitation = 4362), “*Journal of Hepatology*” (cocitation = 4335), and “*Gastroenterology*” (cocitation = 3881). Furthermore, when considering the impact factor, “*Nature Reviews Gastroenterology*” boasts the highest score (IF = 65.10), followed by “*Gastroenterology*” (IF = 29.40). To provide a comprehensive depiction of the cocitation relationships among journals, we set a filter to select journals with a minimum cocitation count of 150 for inclusion in the cocitation network (Figure 5B). As seen in Figure 5B, “*PLOS ONE*” exhibits positive cocitation relationships with journals such “*Hepatology*” and “*Gastroenterology*,” indicating active engagement and cross‐referencing within the field.

**Figure 6:**
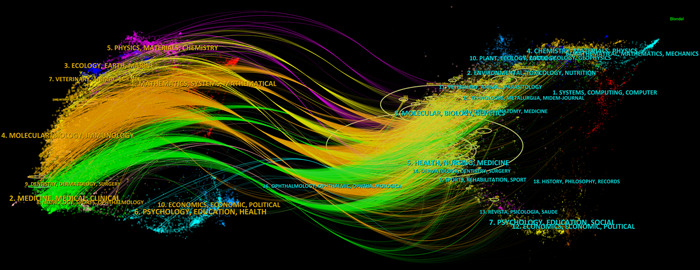
The dual‐map overlay of journals on the research of mitochondria in NAFLD. NAFLD, nonalcoholic fatty liver disease.

The application of CiteSpace has considerably simplified the process of creating a dual‐map overlay, which serves as a valuable instrument to delve into the disciplinary associations within the research landscape. Within this dual‐map overlay, the left and right sections correspond to the journals that are citing and cited, respectively. These sections are interconnected by waves of citations.^20^ The citing side can be considered as the forefront of ongoing research, whereas the cited side represents the fundamental knowledge of which this research is built upon (Figure 6).

In this analysis, we can observe the most significant and influential areas of study, prominently featuring disciplines such as “Molecular Biology” and “Immunology,” “Veterinary” and “Animal Science,” “Medicine,” “Medical,” “Clinical,” as well as “Physics,” “Materials,” and “Chemistry.” The majority of citations lead toward two central fields: “Molecular Biology” and “Genetics,” and “Health,” “Nursing,” “Medicine.” Moreover, disciplines such as “Environmental,” “Toxicology,” and “Nutrition,” along with “Physics,” “Materials,” and “Chemistry,” have also garnered substantial attention. Figure 6 provides a visual representation, with the orange path indicating the primary trajectory of citations and highlighting the predominant flow of scholarly influence.

### 3.4 Authors and cocited authors

**Figure 7:**
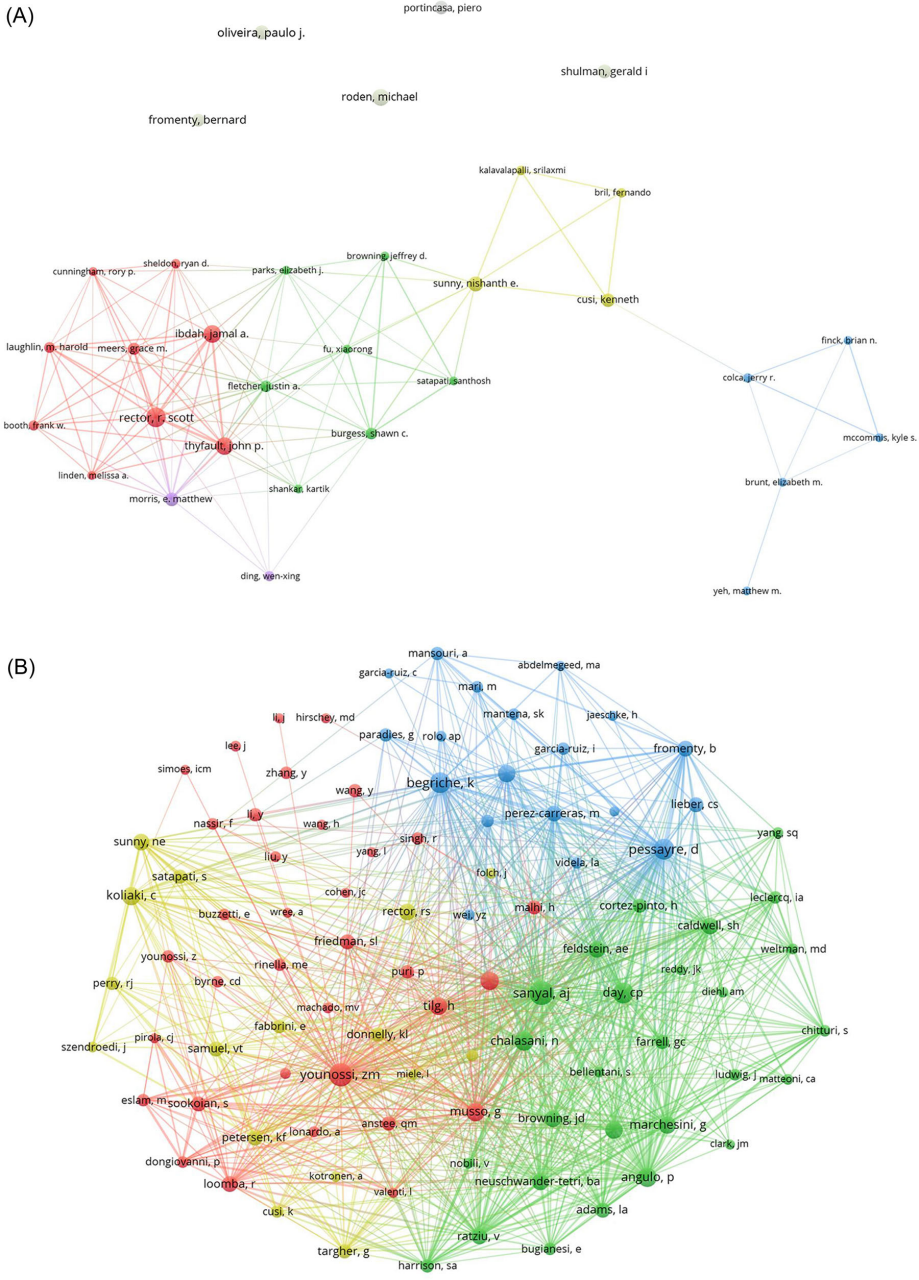
The visualization of authors (A) and cocited authors (B) on the research of mitochondria in NAFLD. The node and line color represented the cluster it belonged to. NAFLD, nonalcoholic fatty liver disease.

A comprehensive analysis reveals that a staggering number of 14,543 researchers have actively engaged in studying mitochondria with NAFLD. Interestingly, the top 10 contributors in this area display remarkable dedication, with an astounding seven of them having made substantial contributions by authoring more than 20 papers each (as depicted in Table 3). To better understand the collaborative dynamics within this field, we have constructed an extensive network representation, encompassing authors who have published six or more papers in this particular domain (as illustrated in Figure 7A). It is worth highlighting that Rector R. Scott, Thyfault John P., and Ibdah Jamal A. stand out prominently in this network, symbolizing their extensive involvement and contributions to this domain of research. Furthermore, it is encouraging to observe synergistic collaborations among several authors. For instance, Rector R. Scott actively collaborates with Thyfault John P., Ibdah Jamal A., and Morris, E. Matthew, while Sunny Nishanth E. engages in active cooperation with Cusi Kenneth, among others.

**Table 3:** Top 10 authors and cocited authors on the research of mitochondria in NAFLD.

Rank	Authors	Count	Cocited authors	Citations
1	Roden, Michael	35	Samyal, A. J.	589
2	Rector, R. Scott	28	Younossi, Z. M.	560
3	Portincasa, Piero	24	Pessayre, D.	451
4	Thyfault, John P.	23	Begriche, K.	443
5	Oliveira, Paulo J.	23	Day, C. P.	441
6	Shulman, Gerald I.	23	Angulo, P.	389
7	Ibdah, Jamal A.	22	Chalasani, N.	369
8	Fromenty, Bernard	18	Musso, G.	344
9	Sunny, Nishanth E.	16	Koliaki, C.	339
10	Cusi, Kenneth	14	Kleiner, D. E.	338

Abbreviation: NAFLD, nonalcoholic fatty liver disease.

Within the expansive pool of 64,537 cocited authors, an esteemed group of 97 authors has amassed cocitations exceeding 100 times (refer to Table 3). At the top of this list is Sanyal A. J. (*n* = 589), followed closely by Younossi Z. M. (*n* = 560) and Pessayre D. (*n* = 451). Authors who have garnered a minimum cocitation count of 100 were carefully selected to construct cocitation network graphs (refer to Figure 7B). As exemplified in Figure 7B, vibrant collaborations are discernible among a diverse assortment of cocited authors, with noteworthy partnerships being evident between Younossi Z. M. and Sanyal A. J., as well as Pessayre D. and Day C. P.

### 3.5 Cocited references

**Figure 8:**
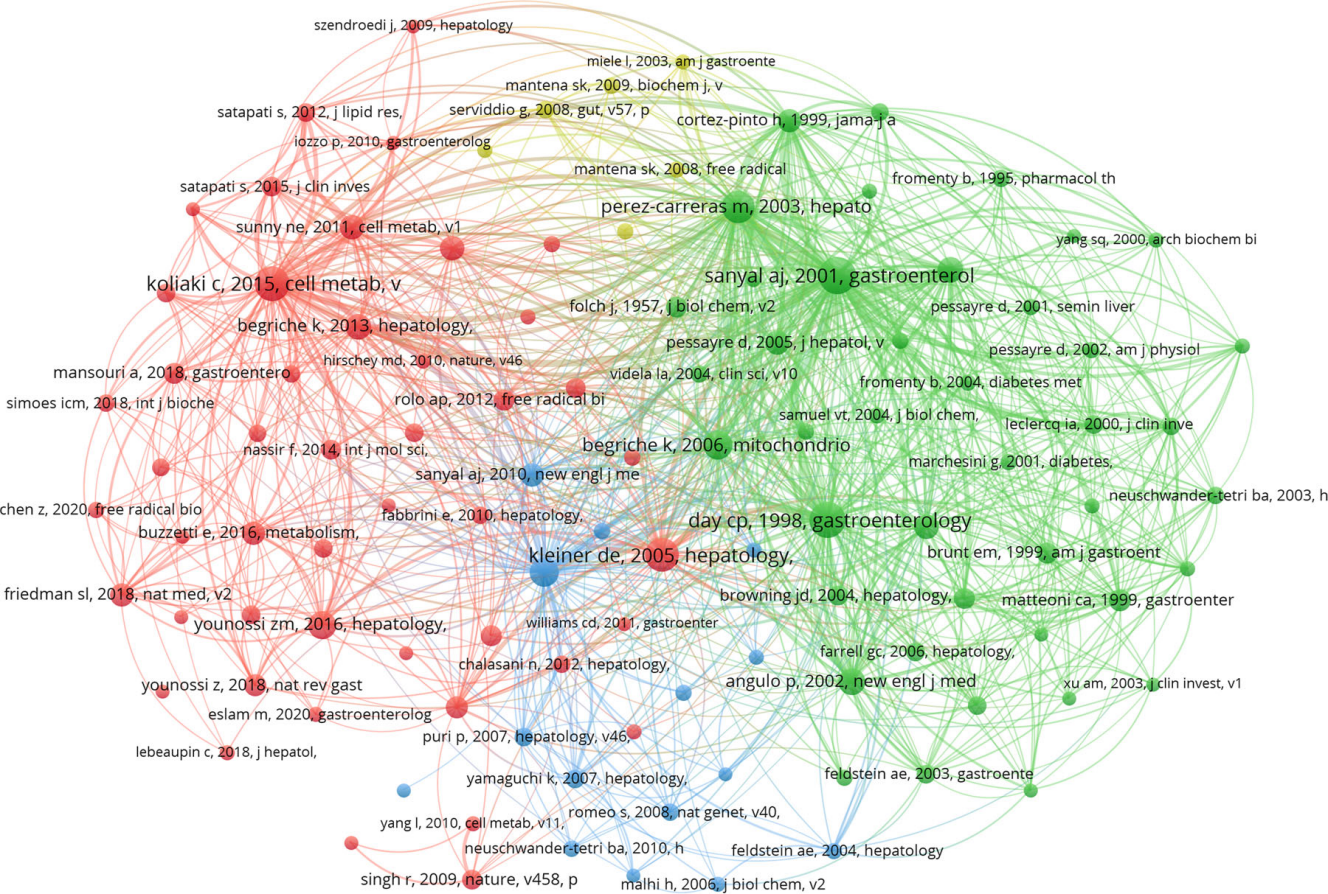
The visualization of cocited references on the research of mitochondria in NAFLD. The node and line color represented the cluster it belonged to. NAFLD, nonalcoholic fatty liver disease.

In the past 20 years, an extensive collection of cocited literature regarding mitochondria in NAFLD research has emerged, comprising a staggering total of 104,090 references. Among the top 10 cocited references (as outlined in Table 4), each reference has garnered no less than 170 cocitations, with two references surpassing a remarkable 300 citations.^20^ To construct the cocitation network map (Figure 8), we specifically chose references that had received 50 or more cocitations. Figure 8 aptly illustrates how “Koliaki C., 2015, *Cell Metab*” shares active cocitation relationships with significant references such as “Kleiner D. E., 2005, *Hepatology*,” “Younossi Z. M., 2016, *Hepatology*,” and “Friedman S. L., 2018, *Nat Med*,” among others.

**Table 4:** Top 10 authors and cocited authors on the research of mitochondria in NAFLD.

Rank	Cocited reference	Citations
1	Sanyal AJ, 2001, *Gastroenterology*, v120, p1183^21^	338
2	Day CP, 1998, *Gastroenterology*, v114, p842^22^	320
3	Koliaki C, 2015, *Cell Metab*, v21, p739^23^	296
4	Kleiner DE, 2005, *Hepatology*, v41, p1313^24^	293
5	Perez‐Carreras M, 2003, *Hepatology*, v38, p999^25^	270
6	Begriche K, 2006, Mitochondrion, v6, p1^26^	212
7	Donnelly KL, 2005, *J Clin Invest*, v115, p1343^27^	210
8	Younossi ZM, 2016, *Hepatology*, v64, p73^28^	202
9	Angulo P, 2002, *New Engl J Med*, v346, p1221^29^	186
10	Begriche K, 2013, *Hepatology*, v58, p1497^30^	171

Abbreviation: NAFLD, nonalcoholic fatty liver disease.

These findings accentuate the notable prevalence of cocited literature revolving around mitochondrial research within the domain of NAFLD, with particular emphasis on the most frequently cited references that substantially shape this field.

### 3.6 Reference with citation bursts

**Figure 9:**
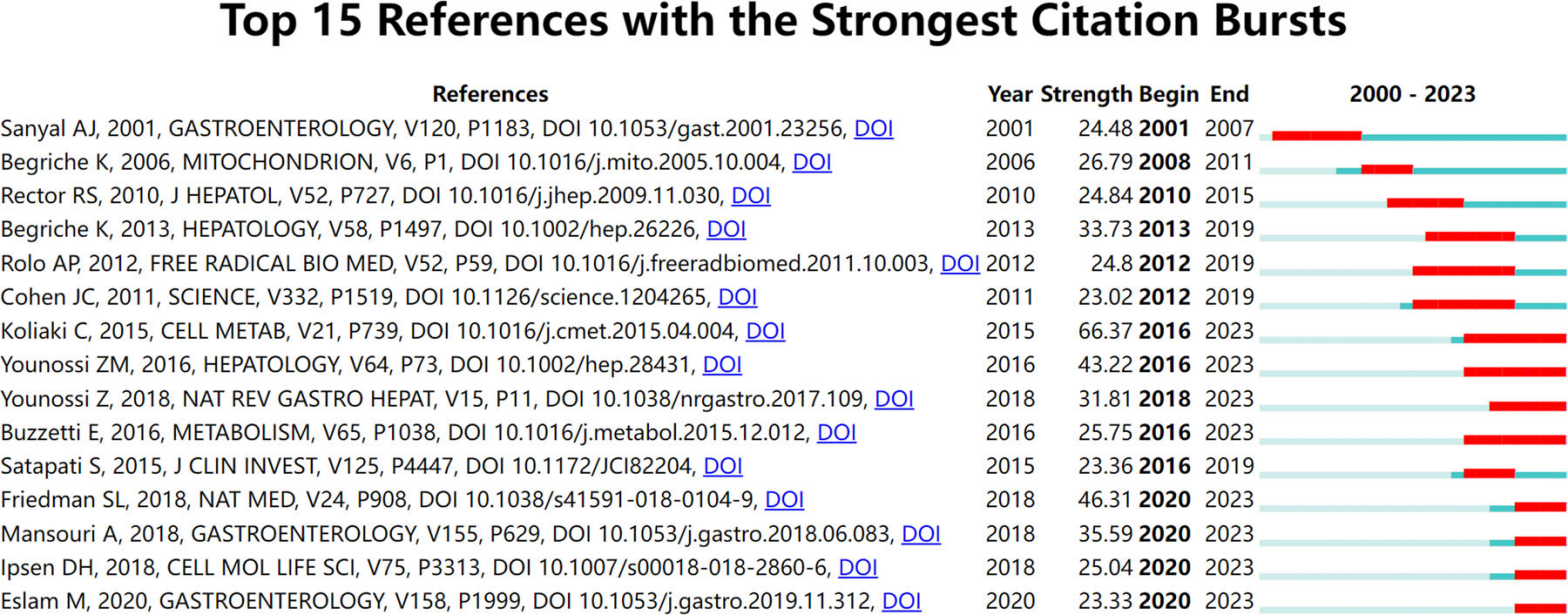
Top 15 references with strong citation bursts. A red bar indicates high citations in that year.

In the field of academic research, the concept of “reference with citation bursts” refers to specific references that consistently receive citations over an extended period in a particular academic discipline. In our investigation, we utilized CiteSpace, a research tool, to identify and evaluate references that displayed strong citation bursts, as illustrated in Figure 9. This visual representation showcases the notable red bars that symbolize the citation bursts, ranging from 2001 to 2023. Among these references, one stood out with exceptional prominence—a paper titled “Adaptation of Hepatic Mitochondrial Function in Humans with Nonalcoholic Fatty Liver is Lost in Steatohepatitis,” authored by Chrysi Koliaki et al. This paper's citation bursts persisted from 2016 to 2023. Another cited study titled “Mechanisms of NAFLD Development and Therapeutic Strategies,” authored by Scott L. Friedman et al. and published in the reputable journal NAT MED has demonstrated the second most prominent citation burst (strength = 46.31) observed from 2020 to 2023. The collective strength of citation bursts for these 15 references ranged from 23.02 to 66.37, spanning a duration of 4−8 years. To provide a comprehensive overview, the primary research themes of these 15 references are presented in Table 5, corresponding to their respective positions in Figure 9. This analysis offers valuable insights into the academic landscape surrounding these influential references within our field of study.

**Table 5:** The main research contents of the 15 references with strong citations bursts.

Rank	Strength	Main research content
1	24.48	Peripheral insulin resistance, increased fatty acid beta oxidation, and hepatic oxidative stress are present in both fatty liver and NASH, but NASH alone is associated with mitochondrial structural defects.^22^
2	26.79	Mitochondrial dysfunction in NASH: causes, consequences, and possible means to prevent it.^26^
3	24.84	Hepatic mitochondrial dysfunction precedes the development of NAFLD and insulin resistance in the OLETF rats. This evidence suggests that progressive mitochondrial dysfunction contributes to the natural history of obesity‐associated NAFLD.^31^
4	33.73	A progressive reduction of mitochondrial respiratory chain (MRC) activity during NAFLD, which could impair energy output and aggravate ROS overproduction by the damaged MRC.^30^
5	24.8	Lipid accumulation in hepatocytes leads to increased production of reactive oxygen species (ROS) and reactive aldehyde derivatives, resulting in oxidative stress and cell death through ATP, NAD, and glutathione depletion as well as DNA, lipid, and protein damage.^32^
6	23.02	The pathogenesis of hepatic steatosis in humans; relationship between obesity, insulin resistance, and hepatic steatosis; genetic risk factors for hepatic steatosis.^33^
7	66.37	Adaptation of hepatic mitochondrial function in humans with nonalcoholic fatty liver is lost in steatohepatitis.^23^
8	43.22	The economic and clinical burden of nonalcoholic fatty liver disease in the United States and Europe, which will likely increase as the incidence of NAFLD continues to rise.^34^
9	31.81	Global burden of NAFLD and NASH: trends, predictions, risk factors, and prevention.^35^
10	25.75	The “multiple hit” hypothesis considers multiple insults acting together on genetically predisposed subjects to induce NAFLD and provides a more accurate explanation of NAFLD pathogenesis.^36^
11	23.36	Induction of biosynthesis via hepatic anaerobic/aerobic pathways is energetically supported by elevated oxidative metabolism and thus contributes to oxidative stress and inflammation during NAFLD.^37^
12	46.31	Review the pathogenic and clinical features of NAFLD, its major comorbidities, clinical progression, and risk of complications and in vitro and animal models of NAFLD.^38^
13	35.59	Mitochondrial dysfunction and signaling in chronic liver diseases.^39^
14	25.04	Molecular mechanisms of hepatic lipid accumulation in nonalcoholic fatty liver disease.^40^
15	23.33	NAFLD does not reflect current knowledge, and metabolic (dysfunction) associated fatty liver disease “MAFLD” was suggested as a more appropriate overarching term.^41^

Abbreviations: ATP, adenosine triphosphate; NAFLD, nonalcoholic fatty liver disease; NASH, nonalcoholic steatohepatitis.

### 3.7 Hotspots and frontiers

Keyword cooccurrence analysis is a valuable tool for quickly identifying research focal points within a specific field. In our study of mitochondria in NAFLD, Table 6 presents the top 18 high‐frequency keywords. Interestingly, “obesity” and “insulin resistance” emerge prominently, each appearing in over 170 instances, indicating their central role in the investigation of mitochondria in NAFLD.

**Table 6:** Top 18 high‐frequency keywords.

Rank	Keywords	Counts	Rank	Keywords	Counts
1	Nonalcoholic fatty liver disease	244	10	Fatty liver	110
2	Nonalcoholic fatty liver disease	196	11	Lipid metabolism	102
3	Obesity	190	12	Nonalcoholic steatohepatitis	100
4	Insulin resistance	173	13	Nonalcoholic steatohepatitis	86
5	NASH	173	14	Metabolic syndrome	82
6	Liver	148	15	Steatohepatitis	72
7	Inflammation	129	16	Hepatic steatosis	71
8	Steatosis	127	17	Autophagy	68
9	Mitochondrial dysfunction	117	18	Apoptosis	66

Abbreviation: NASH, nonalcoholic steatohepatitis.

**Figure 10:**
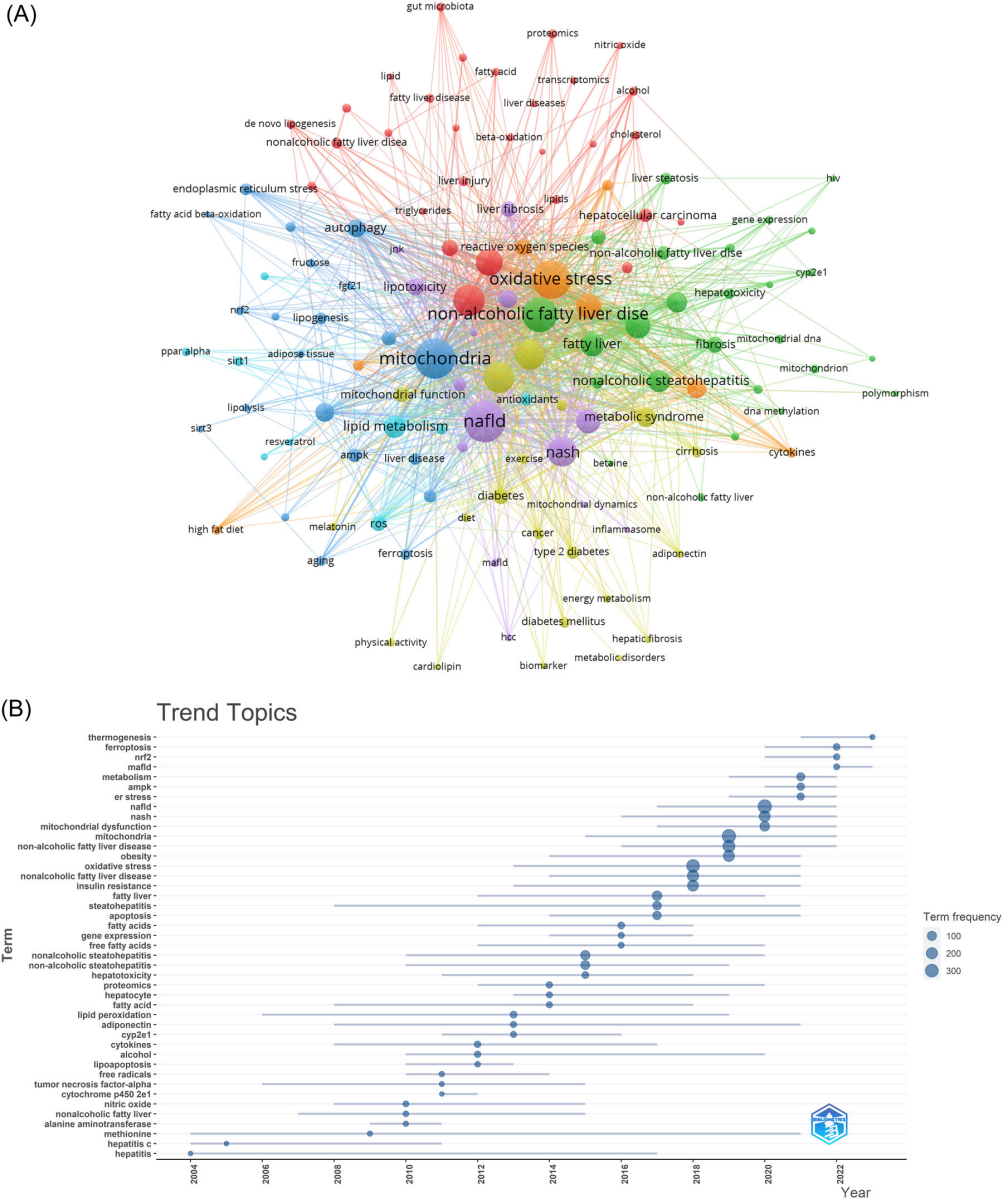
Keyword cluster analysis (A) and trend topic analysis (B) In A, the node and line color represented the cluster it belonged to.

To extract meaningful insights, we filtered keywords with an occurrence count of 10 or more and conducted a cluster analysis using VOSviewer (depicted in Figure 10A). The width of the lines that connect the nodes in the diagram portrays the level of intensity in the connections between the different keywords.

Figure 10A reveals the identification of seven distinct clusters, each representing a unique research direction. The green cluster includes keywords such as “inflammation,” “nonalcoholic fatty liver disease,” “fibrosis,” “nonalcoholic steatohepatitis,” and “fatty liver.” The red cluster revolves around keywords related to “nonalcoholic fatty liver disease,” “liver,” “liver injury,” and “metabolism.” Meanwhile, the blue cluster incorporates keywords associated with “mitochondria,” “autophagy,” “hepatic steatosis,” and related concepts.

The trend analysis of keywords, as depicted in Figure 10B, indicates that from 2000 to 2010, research focused mainly on hepatitis and biochemistry. Key terms dominating this era included “nitric oxide,” “free radicals,” “steatohepatitis,” “hepatitis,” “cytokines,” “lipid peroxidation,” “alanine aminotransferase,” and “methionine.” This period witnessed significant efforts to understand the biochemical aspects of hepatitis, particularly concerning oxidative stress, inflammation, and liver function markers.

Between 2010 and 2015, scholars actively explored the underlying causes, especially obesity, associated with mitochondria involvement in NAFLD. The primary keywords during this period included “fatty acid,” “insulin resistance,” “obesity,” “gene expression,” “hepatocyte,” “apoptosis,” and “free fatty acid,” among others. This phase marked a significant emphasis on unraveling the intricate mechanisms of obesity within the context of NAFLD.

Subsequently, since 2016, scholars have shifted their attention toward investigating the microscopic molecular mechanisms governing mitochondria in NAFLD. In this phase, the central keywords encompassed “mitochondrial dysfunction,” “metabolism,” “thermogenesis,” “ferroptosis,” and related concepts. This transition demonstrates a heightened interest in understanding the finer molecular details of mitochondrial involvement in NAFLD.

Furthermore, within the academic community, there has been a notable surge in interest regarding three pivotal keywords over the course of the last 3 years (2020−2023). These keywords, specifically “ER stress,” “MAFLD,” and “thermogenesis,” have captured considerable attention. Their frequent appearance in current research suggests that they likely represent the prevailing research hotspots in the field of mitochondria in NAFLD. These emerging themes indicate the evolving trends and research priorities in this dynamic area of study.

## 4 DISCUSSION

### 4.1 General information

Within our study, we meticulously collected data from 2530 scholarly papers on the topic of mitochondria in NAFLD over the past 23 years. These publications were sourced from the SCIE database. To gain valuable insights and identify trends, we utilized various bibliometric analyses and data visualization tools, including the Biblimetrix (4.3.1) R package, CiteSpace (6.2.R4), and VOSviewer.

One noteworthy finding is the remarkable growth in the number of publications per year, particularly after 2009. During the period from 2000 to 2008, there was a relatively slow increase in annual publications, reaching a peak of only 50 papers per year. This phase indicated that research on mitochondria in NAFLD was still in its early stages, with a weak research foundation and unclear connections. However, in the subsequent decade from 2009 to 2019, there was a substantial surge, with an average annual publication rate of 113.8 papers. Over the past 4 years, there has been a rapid increase in the number of related publications, suggesting an explosive phase in the study of mitochondria in NAFLD that has attracted significant attention from scholars.

When examining the global landscape, it becomes evident that China and the United States have emerged as significant contributors to research in the field of mitochondria in NAFLD, with the United States holding the leading position. It is worth noting that approximately 60% of the top 10 research institutions are located in China, followed by the United States (30%), and Portugal (10%). A more comprehensive analysis reveals a closely intertwined collaborative network between four countries: the United States, China, Japan, and South Korea. Moreover, the United States actively engages in partnerships with research institutions in Italy, Spain, and Germany, indicating a widespread exchange of knowledge and expertise in this particular arena.

When examining research institutions, we have observed positive collaborative relationships between certain entities, such as the Chinese Academy of Sciences, Peking University, and Harvard Medical School. However, it is worth noting that Chinese institutions tend to prioritize domestic collaborations rather than international exchanges. This domestic‐centric approach may have potential drawbacks for the long‐term advancement of academic research, as international cooperation often brings diverse perspectives and expertise to the table.

Moreover, while there currently exist cooperative relationships among nations, the level and breadth of collaboration among institutions are suboptimal. For instance, the partnership between European institutions and their Chinese counterparts is significantly limited. Such a situation can potentially impede the long‐term advancement of research in the field. Consequently, we strongly advocate for research institutions from diverse countries to actively engage in extensive cooperation and effective communication, thereby working together to collectively propel the study of mitochondria in NAFLD to new heights. These collaborative endeavors possess the potential to enrich the research landscape and yield more profound impacts, benefiting not only the global scientific community but also catalyzing progress in this domain.

The publishing landscape surrounding NAFLD and its connection to mitochondria offers valuable insights into the prevailing trends. A significant majority of the research conducted in this particular field finds its home in the “*International Journal of Molecular Sciences*.” With an impressive impact factor of 5.60 and an unrivaled status as the most popular journal in this research domain, its significance as a platform for disseminating knowledge and findings related to mitochondria in NAFLD cannot be overstated.

Amongst the top 15 journals contributing to this body of research, the standout is the renowned “*Journal of Hepatology*,” boasting an impressive impact factor of 25.70. Closely trailing behind is another journal, “*Hepatology*,” with an impact factor of 17.29. The prominence of these journals as conduits for disseminating research findings serves as a testament to the high‐quality scholarship that characterizes the field. Furthermore, an analysis of cocited publications reveals that eight out of the top 10 journals are prestigious Q1 journals with high impact factors, further attesting to the robust quality of the publications within this area. Undoubtedly, these journals are paramount in providing a solid foundation for advancing research on mitochondria in NAFLD.

Moreover, a closer examination of the current research landscape suggests a strong presence of publications in the fields of Molecular Biology and Immunology. However, an absence of studies in clinically focused journals is apparent. This observation suggests that the majority of ongoing research in this field remains rooted in basic scientific exploration. While these foundational studies are crucial in building a comprehensive understanding of the topic, they also emphasize the potential for future translational endeavors to bridge the gap between basic science and clinical applications in NAFLD and mitochondrial research.

Our research reveals the various stages of evolution in the field, with the years from 2001 to 2012 representing the early stages. During this time, the focus of research was primarily on establishing fundamental theories to serve as the basis for further investigations. To illustrate, in 2001, Sanyal A. J.'s pioneering work illuminated the connection between NASH and abnormalities in mitochondrial structure, laying the groundwork for extensive research into the impact of mitochondrial deletions on the development of NAFLD.^21^ Similarly, in 2006, Karima Begriche's findings highlighted the crucial role of mitochondrial dysfunction, specifically respiratory chain deficiency, in the pathophysiology of NASH.^26^ This discovery emphasized that many drugs capable of preventing or reversing NASH exert their effects by improving liver mitochondrial function, such as metformin,^42^ thiazolidinediones (TZDs),^43^ and fibrates.^44^ Overcoming oxidative stress and mitochondrial dysfunction continues to be a significant challenge for the forthcoming decade.

Moreover, a study conducted by R. Scott Rector in 2010 revealed that hepatic mitochondrial dysfunction occurs before the manifestation of NAFLD and IR.^31^ This research emphasized the role of progressive mitochondrial dysfunction in the natural progression of NAFLD, particularly in individuals who are obese. In a similar vein, Anabela P. Rolo's findings in 2012 shed light on the detrimental impact of lipid accumulation within hepatocytes on mitochondrial oxidative capacity.^32^ This impairment triggers the activation of peroxisomal and microsomal lipid oxidation pathways. Consequently, this process leads to an increase in ROS and reactive aldehyde derivatives, thereby instigating oxidative stress, cellular damage, and inflammation. Ultimately, this cascade of events serves as a driving force behind the development of NASH and potentially end‐stage liver disease.

These groundbreaking studies conducted at different time periods collectively lay a solid foundation for our current understanding of the pivotal role played by mitochondria in the development of NAFLD. During the subsequent phase of exponential growth in publication, scholars in this particular field directed their focus towards exploring the complex mechanisms and signaling pathways that govern mitochondria in NAFLD. In addition, they expanded their investigations to encompass the involvement of mitochondria in various liver diseases and cellular interactions.

For instance, in the year 2020, Mohammed Eslam and his team reached a consensus, acknowledging that the term “NAFLD” no longer accurately encapsulates the current state of knowledge.^41^ Instead, they proposed “Metabolic (Dysfunction)‐Associated Fatty Liver Disease” (MAFLD) as a more suitable overarching term. This shift in terminology provided an opportunity for the research community to update and further categorize the disease, thus accelerating progress toward translational treatments. Furthermore, in the same year, Kapil K. Upadhyay made a groundbreaking discovery by demonstrating the potential of CO‐releasing molecules A1 （CORM‐A1） to mitigate tissue damage in steatotic livers.^45^ This effect was achieved through the activation of Nrf2 and the improvement of mitochondrial function, suggesting the promising potential of CORM‐A1 as an anti‐NAFLD agent.

Building upon these insights, the research conducted by Tao Zhang in 2023 uncovered a range of abnormal mitochondrial functions that contribute to NAFLD.^7^ These include reduced electron transport chain (ETC) activity, impaired adenosine triphosphate (ATP) synthesis, excessive production of ROS, suppressed mitochondrial biogenesis, compromised mitochondrial dynamics, impaired mitophagy, and dysfunction of mitochondrial‐associated membranes.

In summary, recent findings strongly indicate a close connection between NAFLD and mitochondrial dysfunction, emphasizing the emerging perspective that NAFLD can be considered as a core mitochondrial disease.

When examining the cocited authors in the field, it becomes apparent that Sanyal A. J. has emerged as the most prominently referenced author, boasting an impressive count of 589 citations. Hot on his heels are Younossi Z. M., with a substantial 560 citations, and Pessayre D., with a commendable 451 citations. Their contributions have proven to be instrumental in advancing our understanding of the role of mitochondria in NAFLD.

In 2001, Sanyal A. J. conducted a seminal study that advanced our comprehension of the disease.^21^ By examining mitochondrial structural abnormalities in NASH patients and those with fatty liver, the study revealed a strong association between NASH and mitochondrial defects, marking a crucial discovery. In subsequent research in 2008, Sanyal A. J. identified a link between NASH and hepatocyte mtDNA depletion, shedding light on the disease's pathogenesis.^46^ His work in 2015 further elucidated the role of mitochondrial dysfunction in NAFLD, emphasizing impaired ATP production and disrupted fatty acid catabolism.^47^

Bibliometrics have developed in the medical sector in recent years, and they have been demonstrated to offer insights into novel themes as well as retrospective reviews of previous research patterns. For instance, the first comprehensive bibliometric study of studies on mitophagy in liver diseases was carried out by XiuJun Cai's group, which improved our knowledge of the connection between mitochondria and liver disease.^48^

### 4.2 Hotspots and frontiers

References with citation bursts serve as vital indicators of emerging topics within a specific research field, as these references have garnered frequent citations from researchers in recent years.^49^ Examining the primary research themes found within references experiencing robust citation bursts, as detailed in Table 5, highlights that the current focal points in mitochondria research within the context of NAFLD revolve around understanding the biological role and pathogenesis of mitochondria in NAFLD, as well as exploring how these mechanisms can be harnessed for NAFLD treatment. These mechanisms encompass a range of aspects, including alterations in mitochondrial ROS formation and ROS‐signaling pathways, changes in mitochondrial biogenesis and mitophagy, and modifications in mitochondrial levels of cholesterol and GSH (glutathione), as well as in FFAs (free fatty acids), lipid peroxidation products, and TNF (tumor necrosis factor). These areas of investigation collectively represent the forefront of research in the field, emphasizing the importance of unraveling the intricate connections between mitochondrial function, oxidative stress, and the pathogenesis of NAFLD, with the ultimate goal of translating these insights into innovative approaches for NAFLD treatment.

Beyond references exhibiting citation bursts, keywords play a crucial role in promptly identifying how hotspots in mitochondria research within the NAFLD field are distributed and evolving. Excluding specific keywords like “nonalcoholic fatty liver disease,” “mitochondrial dysfunction,” and “liver,” Table 6 predominantly lists essential keywords, including “obesity,” “insulin resistance,” “inflammation,” “lipid metabolism,” and “autophagy.” These key areas of investigation shape the forefront of research in the field, underscoring the significance of unraveling the intricate connections between mitochondrial function, oxidative stress, and the pathogenesis of NAFLD. Ultimately, the aim is to translate these insights into innovative approaches for the treatment of NAFLD. Our analysis, based on keyword clustering and trend topic analysis (Figure 10), leads us to conclude that the primary research directions in mitochondria within the NAFLD context revolve around these aspects: obesity, IR, inflammation, mitochondrial dysfunction, and autophagy.

### 4.3 Inflammation

Inflammation plays a crucial and pivotal role in the progression of NAFLD, which is accompanied by increased levels of oxidative stress and the accumulation of lipids in the liver.^50^ A particular group of liver cells known as Kupffer cells, constituting around 10% of all liver cells, significantly contribute to the inflammatory response within the liver. These specialized macrophages are well‐known for their ability to produce proinflammatory cytokines, which play a critical role in activating T cells and regulating the apoptosis of hepatocytes.^51^ In the context of NAFLD, Kupffer cells display two distinct phenotypes: the proinflammatory M1 cells and the anti‐inflammatory M2 cells.

Under the influence of proinflammatory factors such as interferon‐gamma (IFNγ) and toll‐like receptor (TLR) ligands, M2 macrophages undergo a process known as classical activation, which leads them to transition into proinflammatory M1 cells.^52^ This transition initiates the production of inflammatory cytokines, including TNF‐α and various chemokines. Chemokines belong to a family of cytokines that are involved in the chemotaxis of leukocytes, and their presence contributes to the development of inflammation as well as secondary IR.

To counteract this inflammation, IL‐4 and IL‐13 act as stimulants for M1 cells to transform into anti‐inflammatory M2 cells. These M2 cells play a vital role in neutralizing the proinflammatory cells through apoptosis (cell death). The maintenance of a delicate balance between M1 and M2 cells is crucial for the regulation of liver inflammation.^53^ However, as the progression of NAFLD occurs, this balance tends to shift toward a dominance of M1 cells.

### 4.4 Mitochondrial dysfunction

Mitochondria are highly significant cellular organelles responsible for energy storage and fatty acid metabolism, making them crucial for maintaining energy homeostasis.^54^ They accomplish this through processes such as β‐oxidation, ETC functioning, and the production of ATP and ROS. Disturbances in mitochondrial function, particularly in the ETC and beta‐oxidation pathways, have long been associated with NAFLD. These disturbances lead to inadequate fatty acid oxidation, elevated ROS levels, and increased oxidative stress, all of which contribute to the progression of NAFLD.^55^ Furthermore, mitochondrial dysfunction triggers signaling pathways that induce necroinflammation in liver cells, exacerbating mitochondrial damage.

The dysfunctional mitochondrial activity observed in NAFLD also prompts the accumulation of FFA in hepatocytes, resulting in IR, and ultimately the development of NAFLD, especially in individuals with a high‐fat diet. Numerous protein families can contribute to mitochondrial dysfunction, one of which includes the sirtuins. Sirtuins, such as SIRT1 and SIRT3, play crucial roles in regulating oxidative stress and fatty acid oxidation. Decreased levels of SIRT3 have been observed in animals with fatty livers, potentially worsening the mitochondrial dysfunction.^56^ Additionally, sirtuins rely on nicotinamide adenine dinucleotide (NAD+) for their activity, and depletion of NAD+ can lead to mitochondrial dysfunction and increased FFA levels in hepatocytes.

Mitochondrial transporters, notably Slc25a1 (citrate carrier) and carnitine are vital for maintaining mitochondrial function and facilitating fatty acid metabolism. Inhibition of Slc25a1 has been linked to decreased steatosis, protection against steatohepatitis, and reduced adipose tissue inflammation.^57^ Carnitine, responsible for transporting long‐chain fatty acids into the mitochondrial matrix, exhibits antioxidant properties, enhances β‐oxidation, and mitigates IR. Supplementation with carnitine has demonstrated improvements in various parameters in patients with NAFLD, including aspartate aminotransferase (AST), alanine aminotransferase (ALT), triglyceride (TG) levels, and the homeostatic model assessment of IR (HOMA‐IR).

The production of ROS by mitochondria necessitates antioxidant activity; however, this can be compromised in NAFLD. Notably, reduced glutathione peroxidase (GPx) activity has been observed in animal models of NASH, likely due to glutathione depletion and impaired transport to the mitochondrial matrix.^58^ Additionally, the C47T polymorphism in the gene encoding superoxide dismutase 2 (SOD2) has been associated with reduced SOD2 activity, elevated ROS levels, and an increased risk of NASH development and advanced fibrosis in NAFLD. These findings underscore the significant link between mitochondrial dysfunction and the progression of fatty liver disease.

### 4.5 Autophagy

Autophagy, a vital cellular process, has been implicated in hepatic diseases and is believed to act as a protective mechanism against the progression of steatosis and fatty hepatitis by preventing harm to liver cells.^59^

Within the liver, autophagy functions as a “recycling mechanism” inside hepatocytes, to salvage essential metabolites and provide energy to support cellular functions. This process is essential in maintaining cellular equilibrium by identifying and targeting dysfunctional cellular components for degradation in lysosomes.^60^ “mTOR,” an abbreviation for “mechanistic target of rapamycin,” is a serine/threonine protein kinase in the PI3K‐related kinase (PIKK) family.^61^ It forms the catalytic subunit of two distinct protein complexes, mTOR Complex 1 (mTORC1) and 2 (mTORC2). Because of its crucial role in lipid metabolism, mTORC1 signaling contributes to the inhibition of liver lipophagy by promoting lipogenesis.^62^ The regulation of autophagy is influenced by various factors, such as amino acids, insulin, and the mTOR signaling pathways, which can either inhibit or promote autophagy. Due to its pivotal role, autophagy assumes a central position in the regulation of liver physiology and the maintenance of hepatic metabolism.

Numerous research studies have been conducted to investigate the impact of inhibiting autophagy‐related genes or promoting autophagy on relieving endoplasmic reticulum (ER) stress and hepatic steatosis, thereby emphasizing its protective role.^63^ Autophagy has also been found to play a role in lipid metabolism by breaking down lipid droplets under normal physiological conditions.^64^ Recent investigations have shed light on the liver X receptor α (LXRα) as a key transcriptional regulator of microRNAs (miRs), with the ability to impede autophagy.^65^ This mechanism has been corroborated in both human samples and animal models with genetic obesity. It is noteworthy that LXRα, alongside the identified molecules, also impairs lipid degradation and mitochondrial function, further strengthening the association between autophagy and NAFLD, particularly when LXRα is activated.^66^ These groundbreaking discoveries offer significant potential for target identification and the development of therapeutic strategies for addressing NASH.

### 4.6 Advantages and limitations

This study offers several significant strengths that set it apart. First, it stands as the pioneering comprehensive bibliometric analysis of research on mitochondria in NAFLD, providing valuable guidance for scholars engaged in similar studies. Second, the utilization of three well‐established bibliometric tools, such as VOSviewer and CiteSpace, enhances the objectivity of our data analysis process. Lastly, the inclusion of bibliometric analysis allows for a more comprehensive understanding of research trends and frontiers, surpassing the limitations of traditional reviews.

However, it is important to recognize and address the limitations of this study. First, the scope of this study did not encompass non‐English literature, potentially introducing bias in the data sources used. Second, the ongoing updates to the WoSCC database may lead to slight data discrepancies. However, overall, we are confident that this study has included the majority of relevant original articles, and any changes in conclusions resulting from the updating of a small number of documents are highly unlikely. Additionally, the recently proposed renaming of NAFLD to MAFLD by the international consensus group poses a challenge, as researchers will need to adapt to this evolving terminology and stay updated with the latest research developments. Future bibliometric studies should consider incorporating these new terms to ensure the completeness of retrieval results. Furthermore, it is vital to acknowledge that the information pertaining to the year 2023, included within this investigation, solely encompasses data up until September 20. This clarification is crucial as the study's designated cutoff date concludes at that specific moment in time.

## 5 CONCLUSIONS

The study of mitochondria with NAFLD holds great potential for research and promising prospects for application. The increasing number of publications on this topic indicates a growing recognition of the importance of mitochondrial research in NAFLD among scholars worldwide. While China and the United States are at the forefront of this field, there is a need for improved collaboration and communication among nations and institutions that are involved in this research.

Investigating the intricate roles played by mitochondria in both the development and progression of NAFLD proves to be of utmost importance, as it not only grants us a comprehensive understanding of the underlying causes of the disease but also allows for the early detection and diagnosis. Moreover, delving further into therapeutic strategies aimed at targeting mitochondria holds immense potential for the advancement of targeted and precise treatments of NAFLD in the future. It should be emphasized that, apart from basic scientific research, an equal emphasis must be placed on the translation of research findings into tangible clinical applications, with a particular focus on utilizing mitochondria for effective management of NAFLD in afflicted patients.

Of particular interest, inflammation, autophagy, and mitochondrial dysfunction have emerged as highly active areas of research. Furthermore, the renaming of NAFLD to MAFLD has gained prominence in recent years and is considered a timely and noteworthy topic.

## AUTHOR CONTRIBUTIONS

*Conception and design*: Feng Jiang. *Administrative support*: Ping Li. *Collection and assembly of data*: Jingqin Hu, Ze Chen, and Yibin Zhou. *Data analysis and interpretation*: Jing Liu and Yuqiang Mi. *Manuscript writing*: Jingqin Hu, Li Wang, and Ping Li. *Final approval of manuscript*: All authors.

## CONFLICT OF INTEREST STATEMENT

The authors declare no conflict of interest.

## Data Availability

The data sets generated during the current study are available in the Web of Science (http://www.webofknowledge.com).
